# Passive case detection of malaria in Ratanakiri Province (Cambodia) to detect villages at higher risk for malaria

**DOI:** 10.1186/s12936-017-1758-3

**Published:** 2017-03-06

**Authors:** Somony Heng, Lies Durnez, Sokny Mao, Sovannaroth Siv, Sochantha Tho, Vanna Mean, Vincent Sluydts, Marc Coosemans

**Affiliations:** 1 National Center for Parasitology, Entomology and Malaria Control, Phnom Penh, Cambodia; 2 Department of Biomedical Sciences, Institute of Tropical Medicine, Antwerp, Belgium; 3 Department of Biology, University of Antwerp, Antwerp, Belgium; 4 Department of Biomedical Sciences, University of Antwerp, Antwerp, Belgium

**Keywords:** Malaria, Incidence, Spatial clustering, Passive case detection, Cambodia

## Abstract

**Background:**

Cambodia reduced malaria incidence by more than 75% between 2000 and 2015, a target of the Millennium Development Goal 6. The Cambodian Government aims to eliminate all forms of malaria by 2025. The country’s malaria incidence is highly variable at provincial level, but less is known at village level. This study used passive case detection (PCD) data at village level in Ratanakiri Province from 2010 to 2014 to describe incidence trends and identify high-risk areas of malaria to be primarily targeted towards malaria elimination.

**Methods:**

In 2010, the Cambodian malaria programme created a Malaria Information System (MIS) to capture malaria information at village level through PCD by village malaria workers and health facilities. The MIS data of Ratanakiri Province 2010–2014 were used to calculate annual incidence rates by *Plasmodium* species at province and commune levels. For estimating the trend at provincial level only villages reporting each year were selected. The communal incidences and the number of cases per village were visualized on a map per *Plasmodium* species and per year. Analysis of spatial clustering of village malaria cases by *Plasmodium* species was performed by year.

**Results:**

Overall, malaria annual incidence rates per 1000 inhabitants decreased from 86 (2010) to 30 (2014). Falciparum incidence decreased (by 79% in 2014 compared to 2010; CI 95% 76–82%) more rapidly than vivax incidence (by 19% in 2014 compared to 2010; CI 95% 5–32%). There were ten to 16 significant spatial clusters each year. Big clusters tended to extend along the Cambodian–Vietnamese border and along the Sesan River. Three clusters appeared throughout all years (2010–2014): one with 21 villages appeared each year, the second shrunk progressively from 2012 to 2014 and the third was split into two smaller clusters in 2013 and 2014.

**Conclusion:**

The decline of malaria burden can be attributed to intensive malaria control activities implemented in the areas: distribution of a long-lasting insecticidal net per person and early diagnosis and prompt treatment. Dihydro-artemisinin piperaquine was the only first-line treatment for all malaria cases. No radical treatment with primaquine was provided for *Plasmodium vivax* cases, which could explain the slow decrease of *P. vivax* due to relapses. To achieve malaria elimination by 2025, priority should be given to the control of stable malaria clusters appearing over time.

**Electronic supplementary material:**

The online version of this article (doi:10.1186/s12936-017-1758-3) contains supplementary material, which is available to authorized users.

## Background

Between 2000 and 2015, 57 out of 106 countries reduced malaria incidence by more than 75% and the target of Millennium Development Goal (MDG) 6: “to have halted and begun to reverse the incidence of malaria” has been achieved. Cambodia is among the eight out of ten countries in the World Health Organization (WHO) Western Pacific Region (WPR) that has reached this target [1]. Because of this substantial progress, the Cambodian Government aims to eliminate all forms of malaria by 2025 [2, 3].

In areas of low transmission, differences in malaria exposure at various spatial scales are commonly observed [4]. In Cambodia, for example, incidence is highly variable at province level [5] but less is known at district, commune or village level. Recently, it was shown that clusters of high species-specific PCR prevalence could be detected when analysing village-level data in the province of Ratanakiri [6].

A focus of malaria transmission is a well-defined locality situated in a currently or former malarious area containing the continuous or intermittent epidemiological factors necessary for malaria transmission [4]. A focus can be currently active or not, such as in a formerly malarious area [7]. A geographical part of a focus where malaria transmission intensity exceeds significantly the average level is called a hotspot of malaria transmission. The centre of a hotspot is a mosquito-breeding site, while its radius is determined by the maximal flight distance of mosquitoes. These hotspots, sometimes less than 1 sq km, provide a reservoir of parasites for mosquitoes to spread the infection seasonally to the wider community [4, 8]. Non-targeted vector control interventions aiming for universal coverage, such as long-lasting insecticidal nets (LLINs) or indoor residual spraying (IRS), have been demonstrated to have a large impact on the malaria burden [9], but have no, or limited, impact in tackling residual transmission caused by early and outdoor-biting vectors [10]. However, besides untargeted control, targeting hotspots using additional control methods, such as larviciding tools to control outdoor-biting vectors and mass drug administration, will probably be required to achieve elimination of malaria [4]. Identifying malaria hotspots [7, 11–24] or clusters of malaria cases at sub-provincial level, such as villages, in space and time in such low transmission areas would help in effectively targeting and developing appropriate malaria control strategies towards malaria elimination.

Incidence data are well suited for determining malaria clusters in low transmission settings [25]. This study, based on malaria passive case detection (PCD) data at village level in the province of Ratanakiri, Cambodia from 2010 to 2014, describes recent trends in malaria incidence rates and identifies high-risk areas of malaria to be primarily targeted towards malaria elimination.

## Methods

### Study area profile

Ratanakiri Province, located in the remote northeast of Cambodia, is a mountainous and hilly area covered by tropical forest, rubber plantation or fallow land. The province borders the provinces of Mondulkiri to the south and Stung Treng to the west and the countries of Laos and Vietnam to the north and east, respectively. It comprises nine administrative districts, 49 communes and 240 main-villages [26]. Some main-villages have annex-villages or satellite-villages, which are informal settlements, often remote or geographically isolated and they are established mainly because of population movement and growth [27]. In 2013 there were 110 annex-villages in Ratanakiri (personal communication with the malaria supervisor of the provincial health district). The province houses a population of 183,699 inhabitants (2013) [28] belonging to ten different minority groups that are mostly subsistence farmers cultivating seasonal crops such as dry rice, cassava and beans on slash-and-burn forest fields [29]. A large number of families possess farms far from their villages and own two houses, one in the village and the other in the farm, often located in the forest where they usually stay during planting and harvesting season [29]. During the rainy season (June–October) some areas are not accessible because of poor road conditions and some places are only accessible by boat and or on foot. In 2013 this province had 11 health centres, 19 health posts and a provincial hospital [26]. These health facilities are under the management of Ratanakiri operational district (OD), which is overviewed by Ratanakiri provincial health department (PHD). Some 136 remote malaria-endemic villages have village malaria workers (VMWs) in place. To install a VMW in a village, the village must be located more than 5 km or a 1-h walk from the nearest public health facility (HF). Two villagers, preferably one male and one female, per village are selected as VMWs through community consensus [30]. The VMWs are trained to diagnose malaria using rapid diagnostic tests (RDT) and to treat uncomplicated cases [5]. Malaria endemicity of this province is among the highest in the country with a peak between August and November) and people who stayed overnight in plot-huts at farms were found to be more at risk of malaria infections [11]. LLINs are the main malaria prevention tool used in the province and a combination of dihydroartemisinin and piperaquine is the anti-malarial used for the treatment of all *Plasmodium* spp.

### Data collection

In 2010 a Malaria Information System (MIS) database was created to collect information from all areas at risk [31]. When required, additional support was provided by the MalaResT project [32], such as per diem and travel costs for OD staff to mentor health centres how to record data. MIS captured all individual malaria cases from both VMWs and HFs (in- and outpatients) obtained through PCD [33]. VMWs report the number of cases tested, both having a negative and positive result, while HF reports contain positive cases only. No information from the private sector was captured by the system. Monthly reports of VMWs and HFs are collected by operational districts. They record information on name and code of district, HF and village of origin, year, month, laboratory test results related to malaria parasite species, death, age group (up to 4, 5–14, 15–49 and 50 years and above), and gender. The data entry was done at operational district level using separate databases for VMWs and HFs and monthly updated data extracts were sent to the National Centre for Malaria Control (CNM) by email where they were automatically entered into the national database. The MIS also recorded number of reports received from each VMW village and HF per year. The person at operational district level was responsible for supervising the HFs and VMWs, checking the quality of data and following up on missing reports.

Only RDT, CareStart™ Malaria, pLDH/HRP2 COMBO (PAN/Pf), was used by VMWs to confirm malaria. This test was also used by health staff at HFs without microscopes or when a microscopist was not available, e.g., at night-time or during holidays. However, the proportion of each tool used at HFs was unknown since it was not recorded in the MIS. The national guidelines for interpretation of RDTs were as follows: tests with no band for control are considered invalid (to be repeated); presence of control band alone is negative for *Plasmodium*; presence of control and *Pf* bands is positive for *Plasmodium falciparum*; a control and PAN bands is *Plasmodium vivax*; and, control, *Pf* and PAN are classified as mixed infections. However, the last case (presence of control, *Pf* and PAN bands) can also be *P. falciparum* alone [34–36].

### Data analysis

The two datasets (VMW and HF) were combined into one to get monthly incidence cases by village/annex-village every year, taking into account the frequency of monthly reporting. Cases that were referred to a HF by a VMW and notified as such were removed from the VMW dataset to avoid double counting.

The average number of monthly reports (to MIS) is calculated for VMW and HF systems and by year. The total number of malaria cases per year and per village was further reported on a map and this regardless of frequency of reporting. Taking into account that not all localities had VMWs, and that HFs might deal with patients coming from localities not covered by them, the total number of positive cases per locality per year is provided if at least one report from a local VMW is available or/and at least one report from any HFs in the province reporting a case from that village. Data were considered to be missing for a locality and a specific year if no report from VMW was available and no report for that specific locality from any HFs in the province. According to annual number of positive cases the reporting villages were classified into eight categories (i.e., villages with number of cases of 0, 1, 2–5, 6–12, 21–50, 51–100, >100, and missing data).

Annual incidence rates per 1000 inhabitants at commune level for all and each specific *Plasmodium* spp were calculated. This calculation used projected annual population by commune as denominator. The population projection was done by multiplying population from Cambodia National Census 2008 by annual growth rate taken from population projections for Cambodia, 2008–2030 for Ratanakiri province (2009: 2.54%, 2010: 2.14%, 2011: 1.96%, 2012: 1.97%, 2013: 1.94%, 2014: 1.90%) [37]. To estimate a general trend of malaria incidence in the province, only villages reporting every year (2010–2014) were considered. To estimate the decrease of malaria incidence, the model used was a mixed-effects negative binomial regression using village as a random effect and year as categorical fixed effect. Each model was verified versus a standard negative binomial regression. The analysis was done in Stata/MP 14.2 (StataCorp. 2015 Stata: Release 14. Statistical Software, College Station, TX, USA). The annual trends of total incidence rates were presented in a graph by *Plasmodium* spp. Each year, the communes were classified into seven categories according to their levels of annual incidence rate (i.e. 0, >0 to <1, 1–50, 51–100, 101–150, >150, and missing data). Data analyses were performed using program R v.3.2.0 [38].

All villages and communes were visualized on a map per *Plasmodium spp* and per year using the categories of case number (for villages) and incidence rates (for communes). This mapping was done in QGIS Desktop 2.12.1.

SaTScan v9.4 64-bit, software for the Spatial Scan Statistics, was used to identify clusters with increased risk of malaria in each year. For this spatial analysis, the program identified clusters by gradually checking multiple circular windows with a variable circle size of maximum 50% of the population at risk [39] based on the census in 2008. The analysis estimated the risk of malaria outside and inside each window under a null hypothesis of equal risk using a discrete Poisson model. Using the default maximum number of replication of 999 Monte Carlo simulations, the window having the maximum likelihood was assigned to be a cluster and a p value was provided. A cluster with p value lower than 0.05 was considered as significant. Spatial clustering analysis of malaria cases (all and by species) was done by year.

The GPS coordinates of the villages were provided by the Cambodian national MIS. During a survey [11], 117 out of 240 villages were checked for their GPS coordinates in the field and compared to the MIS coordinates. Most villages (92%) were in a radius of fewer than 500 m, 2.6% between 0.5 and <1 km, and 4.2% between 1 and 4 km. For only one village, the MIS coordinates did not match all the coordinates observed during the survey. The correct GPS positions were then applied for all 117 villages but remained unchanged for the other 123 villages. For the present study, an overall accuracy of 95% for the GPS positions within a radius of less than 1 km was estimated.

## Results

**Fig. 1:**
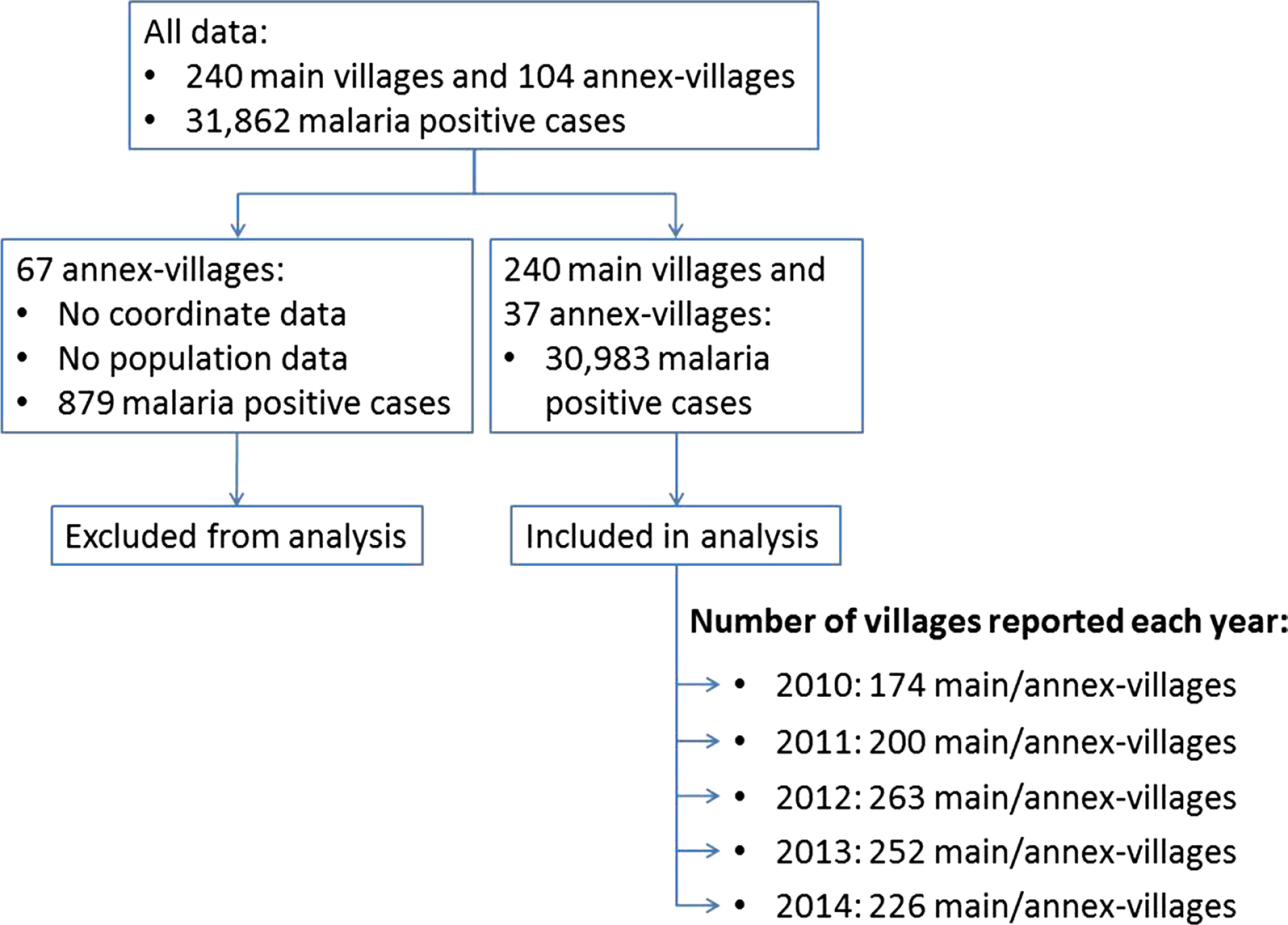
Data exclusion and inclusion. The database is a combination of VMW and HF datasets. Data from 67 annex-villages missing either population or coordinate data were excluded from the analysis. In total, data from 277 (240 main and 37 annex) villages were included in the analysis

A set of VMW and HF data were obtained from MIS of Ratanakiri Province with 31,862 malaria-positive cases reported from 2010 to 2014 from 240 main-villages and 104 annex-villages. Sixty-seven annex-villages with 879 malaria cases (2.8% of the total positive cases) had neither coordinates nor population data, and were therefore excluded from the analysis (Fig. 1). The analysis was conducted on 30,983 cases from 277 villages comprising 240 main- and 37 annex-villages, of which 73.54% (22,786/30,983) were reported by VMWs, diagnosis confirmed by RDT, whereas remaining cases were reported by HFs, confirmed by either microscopy or RDT.

Not all 277 villages delivered reports every year. The percentage of VMW villages delivering 12 monthly reports per year was 22, 98, 94, 87, and 95% in 2010, 2011, 2012, 2013 and 2014, respectively (Additional file 1); of HFs, 8, 15, 90, 97, and 85% delivered 12 monthly reports in 2010, 2011, 2012, 2013 and 2014, respectively (Additional file 2).

### Positive cases and incidence rate

**Fig. 2:**
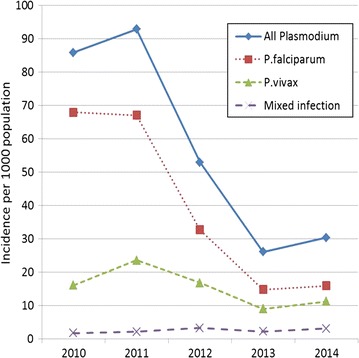
Annual incidence rates per 1000 inhabitants by *Plasmodium* species among 157 villages reporting every year (2010–2014)

Some 157 out of 277 (57%) villages reported every year and were considered for estimating the trend malaria incidence between 2010 and 2014. For these 157 villages the overall annual incidence rates per 1000 inhabitants of all *Plasmodium* spp. decreased dramatically from 85.9 in 2010 to 30.4 in 2014 (Fig. 2). Using a mixed-effects negative binomial regression this decrease was significant from 2012 up to 2014 compared to 2010 (2014 vs 2010: 65%; 95% CI 61–69%). Falciparum cases decreased even more (2014 vs 2010: 79%; 95% CI 76–82%) whereas the incidence for *P. vivax* (2014 vs 2010: 19%; 95% CI 5–32%) and mixed infections fluctuated almost every year (Additional file 3).

**Fig. 3:**
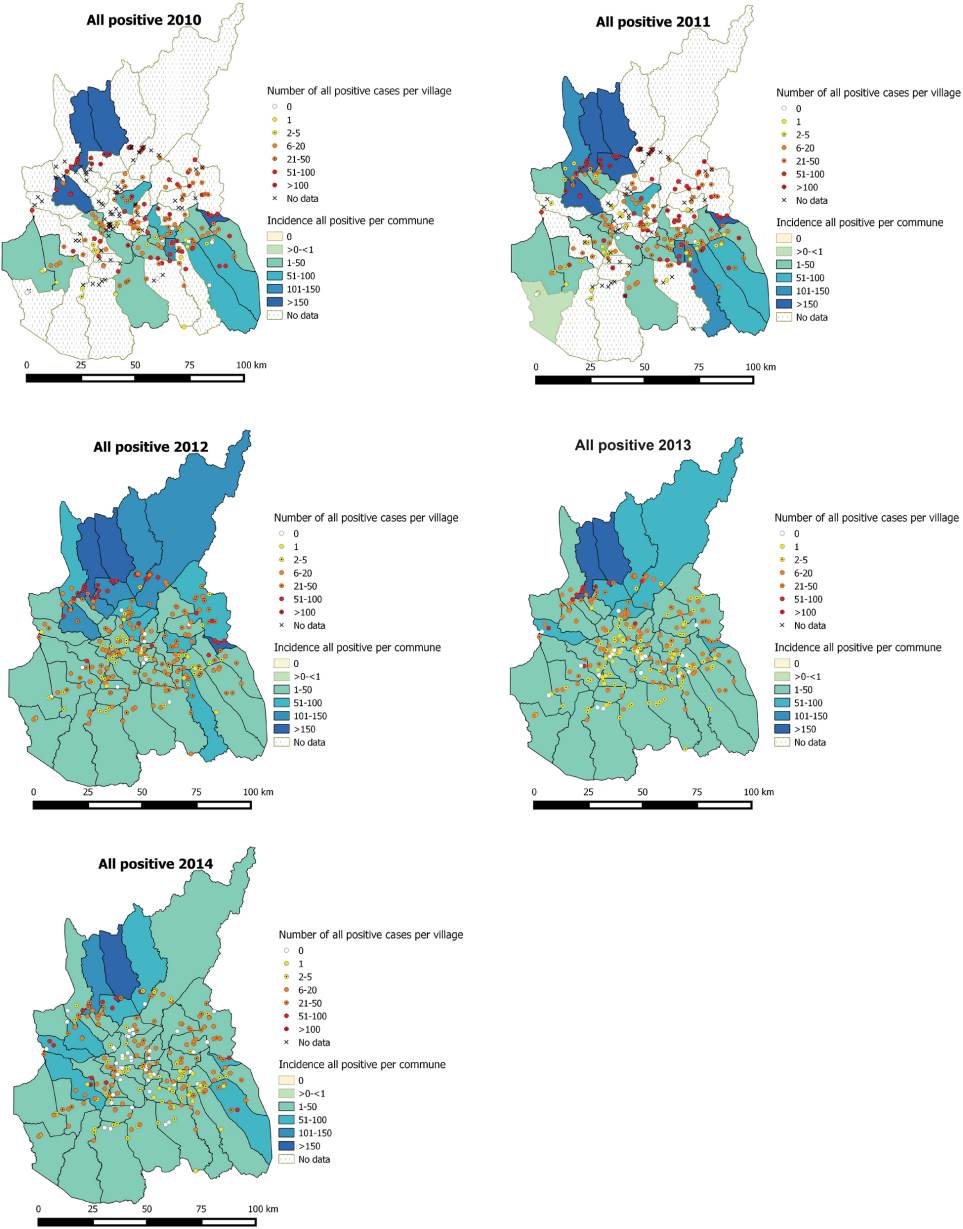
Malaria cases by village and malaria incidence rate per 1000 inhabitants by commune, 2010–2014. Incident cases and incidence rate of all *Plasmodium* species combined. Although several villages did not report in 2010 and 2011 (37 and 28%, respectively, indicated by *X*), the number of confirmed malaria cases and incidence decreased steadily from 2012 to 2014

The number of reporting villages increased from 174 in 2010 to 226 in 2014 (Additional file 4). The number of malaria cases was further reported by village and the incidence was calculated per commune (Fig. 3). Villages with more than 50 malaria cases reported annually remained concentrated at peripheral areas of the province especially along the Sesan River, in the northwest and in the southeast (Fig. 3). This trend looked similar for *P. falciparum* and *P. vivax* separately (Additional files 5 and 6). Since 37 and 28% of villages belonging to 35 and 26 communes in 2010 and 2011, respectively, had no report, the incidence of those communes was not calculated (Fig. 3).

### Malaria cases spatial clustering

**Fig. 4:**
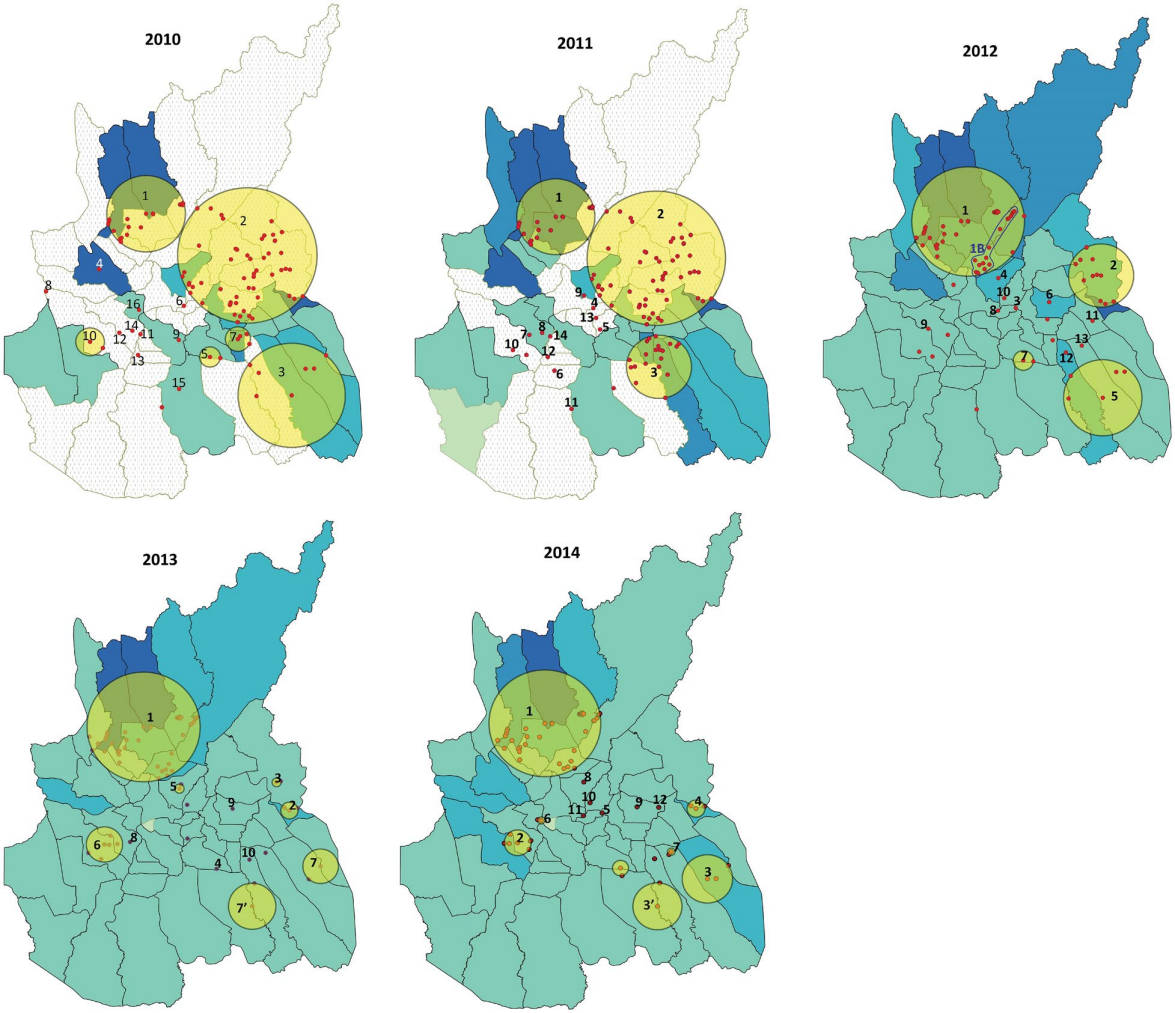
Spatial clusters of all malaria cases. Clusters of villages with higher number of malaria cases detected from 2010 to 2014 in Ratanakiri Province. Each *numbered circle* or *dot* represents a significant cluster. The *un*-*numbered circles* or *dots* are not significant clusters. Incidence of all malaria cases is given per commune

Each year several clusters with a significantly higher risk of malaria cases (all species combined) were found, indicating that malaria is not evenly distributed amongst the province. There were ten to 16 different, significant, spatial clusters each year. The number of significant clusters decreased steadily from 2010 (16) to 2013 (ten) before a slight increase in 2014 (12). Every year the minimum number of villages per significant cluster was one, whereas the maximum varied from 62 in 2010 to 41 in 2014. Detailed information of the significant clusters can be found in Additional file 7. Big clusters tended to extend to the east (along the Cambodian–Vietnamese border) and the north of the province (along the Sesan River). Figure 4 shows that cluster 1 was stable throughout all years and within this cluster there were 21 villages appearing every year and 20 other villages (in the group of village 1B) appeared from 2012 until 2014. Eleven villages of 1B did not report in 2010 and seven villages of this group did not report in 2011. Cluster 2 was shrinking progressively from 2012 to 2014 (2013: clusters 2 and 3; 2014: cluster 4). Cluster 5 observed in 2012 also remained stable from 2012 to 2014, but in 2013 and 2014 it was split into two smaller clusters (2013: clusters 7 and 7′; 2014: 3 and 3′) of three villages.

The distribution of the *P. falciparum* and *P. vivax* clusters were somewhat different although large overlaps were observed (see Additional files 8, 9, 10, 11). In 2010, the large *P. vivax* cluster (cluster 1) has a more western distribution compared to the large *P. falciparum* cluster (cluster 2). In 2013, *P. falciparum* and *P. vivax* clusters overlapped each other. In 2014, a new large *P. vivax* clusters (cluster 4) appeared at the western border of the province.

## Discussion

Estimating malaria incidence is a difficult exercise for control programmes as it relies on accurate and updated census and completeness in reporting malaria cases by health systems. Population size is based on census and further adapted with a national growing rate per year, not taking into account local differences, including migration and temporary population movements. With increased access to malaria diagnosis and treatment, particularly through the VMWs, fewer cases consult the private sector. However data are not available on attendees at private facilities, including traditional healers, which will probably underestimate the burden of the disease [5]. Recognizing these limitations, malaria incidence was calculated for 157 villages reporting each year from 2010 to 2014 and may provide a general trend for the province. The malaria incidence rate of all *Plasmodium* spp combined in these villages steadily declined to a low level, from 73.76 per 1000 inhabitants in 2010 to 25.25 in 2014. Although vivax incidence was less important than falciparum, the latter declined more rapidly especially between 2011 and 2013. This decline can be attributed to the intensive malaria control programme implemented throughout Ratanakiri where one LLIN was distributed per person and access to early diagnosis and prompt treatment was facilitated by a network of VMWs and HFs (according to personal communication with the provincial malaria supervisor, Ratanakiri Province). In 2012 and 2013 topical repellents were distributed in 57 villages for a cluster-randomized trial (MalaResT) but no additional impact of these repellents has been observed on malaria prevalence or incidence [40]. Dihydro-artemisinin piperaquine (DHA–PIP) was the only first-line treatment for malaria of all *Plasmodium* species and no primaquine was used for radical treatment of *P. vivax*. This could explain the slow decrease of *P. vivax* incidence due to relapse cases [41].

The limitation of this study is that only 22% of VMWs reported every month (12 reports per year) in 2010 (Additional file 1), whereas 8 and 15% of HFs reported every month in 2010 and 2011, respectively (Additional file 2). Nevertheless, reporting improved considerably from 2011 (for VMWs) and from 2012 (for HFs) until 2014 with 95% of the VMWs and 85% of HFs reporting every month. For commune level, the incidence rates was based on population size of the 2008 census and corrected with a growth factor by year. However, for village level using absolute number of cases was more appropriate as the population growth factor in each village may be different due to recent population movement, particularly linked to changes in land use, mainly large rubber plantations. Moreover, in the context of elimination where the last malaria case is of importance, control managers are more interested in the absolute number of cases per locality for implementing additional control interventions.

Despite a general decline in malaria burden, the remaining cases were scattered heterogeneously across the province. The relatively low numbers of cases (2.8% of total cases) in the 67 annex-villages not included in the cluster analysis may suggest localities with a relatively low risk of malaria. Including them may have an effect on identifying the clusters. However, it can be assumed that the number of cases is under-reported in these settings as most of these cases (59% or 518 cases) were observed by HFs as no VMWs [5] was present in 96% (64/67) of these new settlements. To omit these cases would be the most conservative approach.

The spatial clustering analysis showed one cluster along the Sesan River re-appearing each year. This cluster was found to be stable throughout all years within the whole focus of Ratanakiri, which is consistent with a finding from another analysis of MalaResT’s survey data from 2012 to 2013 [6]. Within this cluster, 21 villages appeared every year (2010–2014), while 20 other villages appeared in 2012 and remained until the end of the observation period (2014). This might be explained by 11 and seven villages of this village group not reporting in 2010 and 2011, respectively. Similarly, at the Cambodian-Vietnamese border, three villages appeared during three consecutive years (2012–2014) within the same cluster, although the latter was split into two smaller clusters in the later years. These villages, located along Sesan River, were at elevated risk of symptomatic malaria cases (all *Plasmodium* species combined) which confirmed previous observations of clustering of asymptomatic malaria cases in this area [11]. Reasons for this elevated risk of both symptomatic and asymptomatic cases could be that along the Cambodian–Vietnamese border there is uncontrolled cross-border population movement, which coincides with the presence of *Anopheles dirus*, the main outdoor- and early-biting vector [42]. Although a large overlap was seen with several *P. vivax* and *P. falciparum* clusters, some malaria species clusters appeared to be completely isolated (e.g., *P. vivax* cluster 4 in 2014) which corroborates the results of a previous study in the same area [11]. Both malaria species are transmitted by the forest vector *An. dirus* and the river vector *Anopheles minimus*, but *P. vivax* can maintain itself with secondary vectors [50] and relapses [51].

Clusters of malaria cases are not necessarily the same as hotspots of transmission. There may be little or no ongoing transmission in some of the identified clusters of high malaria incidence due to cases imported from elsewhere. Therefore, data on travel history and entomology are needed to confirm potential hotspots. Further investigation should be focused on potential hotspots to explore their specific characteristics and determinants so that targeted and specific interventions could be applied accordingly. Besides these two clusters, the other significant clusters did not occur every year and it can be assumed that some people were infected outside the villages during their occupational travel to hotspots and re-introduced malaria in their village [4].

Besides the mobility of people, the heterogeneity of remaining malaria cases could be due to variable determinants, such as water, lodging, proximity to the forest, population density, area altitude, rainfall, temperature, socio-economic status, hygienic status, distance from sleeping place to livestock holding place and bed net use [15, 17, 20, 21]. Due to this heterogeneity, the efficacy of the current uniform control strategies would be mitigated [4, 11, 22, 23, 43–46]. People living in hotspots are at high risk of malaria infection [47] and malaria there can increase whenever favourable transmission conditions arise [48]. Consequently, these areas become sources of infection transmitting malaria to other places. Targeting intervention to this focal group is likely to have an effect on the entire population [49]. To reach the malaria elimination goal, specific interventions and more research should be focused in villages located in stable clusters (2010–2014) because these villages show continuous and pronounced (in terms of numbers/percentage) presence of malaria cases. This will allow identifying those villages in the cluster with a high probability of hosting an actual hotspot of transmission. The number of main/annex-villages in the significant clusters to be targeted (from the analysis 2014) was 63/277, 23% of all villages, which would be more feasible and cost effective than intervention in the entire province. As Cambodia’s MIS is a government routine system functioning relatively well, the yearly data collection to determine malaria clusters/hotspots would not be too costly. The cluster/hotspot targeted approach would be the best option to speed up malaria elimination in the country.

Knowing the full picture of the malaria burden is important for the national programme to understand the disease situation (by locality and population sub-group) in order to develop appropriate approaches towards malaria elimination [52]. To get a complete picture of malaria incidence, the PCD data should be fully documented and combined with data from all sources. Malaria data are only available from VMWs and public HFs while data from private and military/police sectors are currently not captured in MIS. This should be changed through a public/private mixed channel and inter-sectoral information sharing between military/police and Ministry of Health. Moreover, international donors, particularly the Global Fund, should more focus their support on a performing MIS. Population data and GPS coordinates per village were not always available especially for annex-villages where non-permanent residents or newcomers are predominant and the administrative structure is not always in place. Local health authorities in collaboration with local authorities should take coordinates of all villages and update the population census regularly for use, not only in malaria programmes but also in other health programmes. However, in the pre- and elimination stages, when numbers of cases are declining, capturing absolute numbers of malaria cases per locality is more accurate than estimating an incidence rate relying on incomplete knowledge of population size.

## Conclusions

Malaria burden in the study area had been decreasing steadily, however the remaining cases are distributed heterogeneously across the province, which could mitigate the effectiveness of the current uniform intervention. Clusters of malaria cases and their determinants should be identified annually to look for hotspots to be targeted with specific interventions in order to achieve the malaria elimination goal. Data from private and military/police sectors should be captured in the MIS.

## Additional files

**Additional file 1.** Frequency of village malaria workers reporting per year. One report is expected each month.

**Additional file 2.** Number of health facilities reporting each year and frequency of submission of reports.

**Additional file 3.** Incidence rate ratio with 2010 as reference.

**Additional file 4.** Annual incidence rates per 1000 inhabitants for all reporting villages. The total number of reporting villages was 277 including main- and annex-villages, but not all of them reported every year.

**Additional file 5.** Annual number of confirmed falciparum malaria cases by village and falciparum malaria incidence rate per 1000 inhabitants by commune. Although several villages did not report in 2010 and 2011 (37 and 28%, respectively), the number of confirmed malaria cases and incidence decreased steadily from 2012 to 2014.

**Additional file 6.** Annual number of confirmed vivax malaria cases by village and vivax malaria incidence rate per 1000 inhabitants by commune. Although several villages did not report in 2010 and 2011 (37 and 28%, respectively), the number of confirmed malaria cases and incidence decreased steadily from 2012 to 2014.

**Additional file 7.** Spatial clusters of villages with significantly higher risk of all malaria cases (all *Plasmodium* species combined) from 2010 to 2014 in Ratanakiri Province. Only significant clusters are showed. RR: Relative risk. LLR: Log likelihood ratio.

**Additional file 8.** Spatial clusters of falciparum malaria cases. Clusters of villages with higher number of malaria cases detected from 2010 to 2014 in Ratanakiri Province. Each numbered circle or dot represents a significant cluster. The un-numbered circles or dots are not significant clusters. Incidence of falciparum malaria cases is given per commune.

**Additional file 9.** Spatial clusters of villages with significantly higher risk of falciparum malaria cases from 2010 to 2014 in Ratanakiri Province. Only significant clusters are showed. RR: Relative risk. LLR: Log likelihood ratio.

**Additional file 10.** Spatial clusters of vivax malaria cases. Clusters of villages with higher number of malaria cases detected from 2010 to 2014 in Ratanakiri Province. Each numbered circle or dot represents a significant cluster. The un-numbered circles or dots are not significant clusters. Incidence of vivax cases is given per commune.

**Additional file 11.** Spatial clusters of villages with significantly higher risk of vivax malaria cases from 201 to 2014 in Ratanakiri Province. Only significant clusters are showed. RR: Relative risk. LLR: Log likelihood ratio.

**Additional file 12.** Raw data file. This file contains all raw data used for all analyses in this study.
