# Supplementary figures and images for: Passive case detection of malaria in Ratanakiri Province (Cambodia) to detect villages at higher risk for malaria
Source: Malar J. 2017 Mar 6;16:104. doi: 10.1186/s12936-017-1758-3 (PMC5340042; doi:10.1186/s12936-017-1758-3)

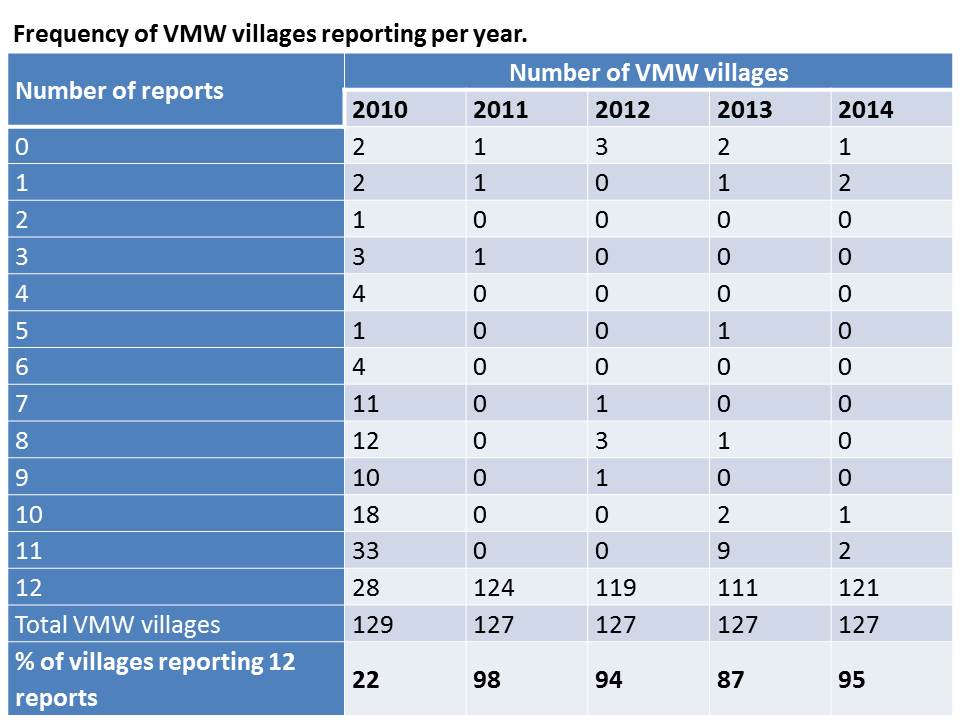

Supplement: Supplementary file 1 — Additional file 1. Frequency of village malaria workers reporting per year. One report is expected each month. [file 12936_2017_1758_MOESM1_ESM.jpg]

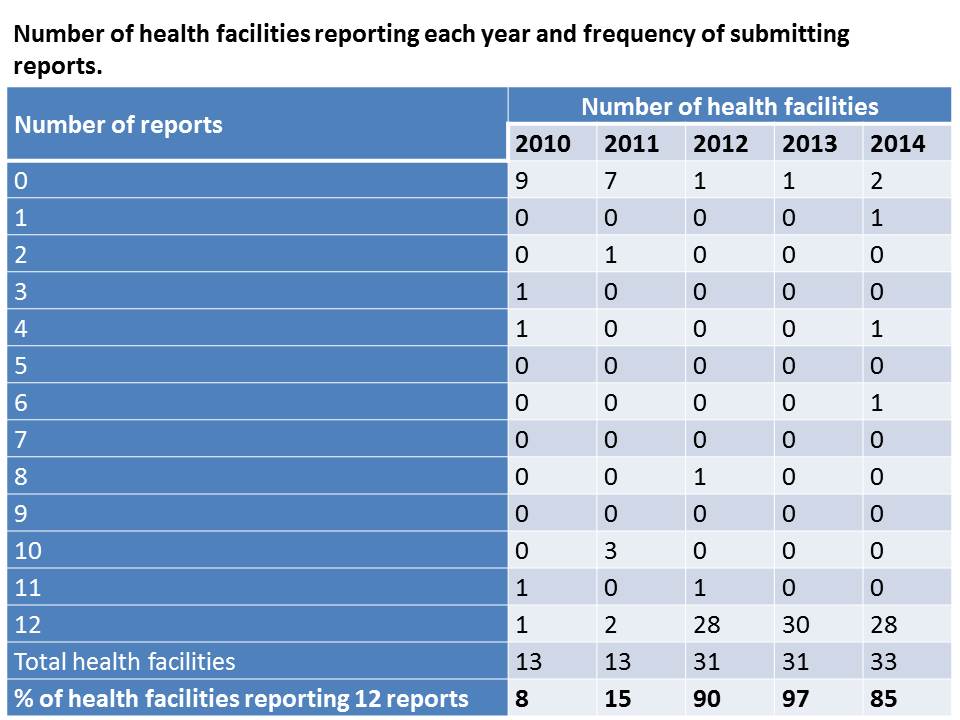

Supplement: Supplementary file 2 — Additional file 2. Number of health facilities reporting each year and frequency of submission of reports. [file 12936_2017_1758_MOESM2_ESM.jpg]

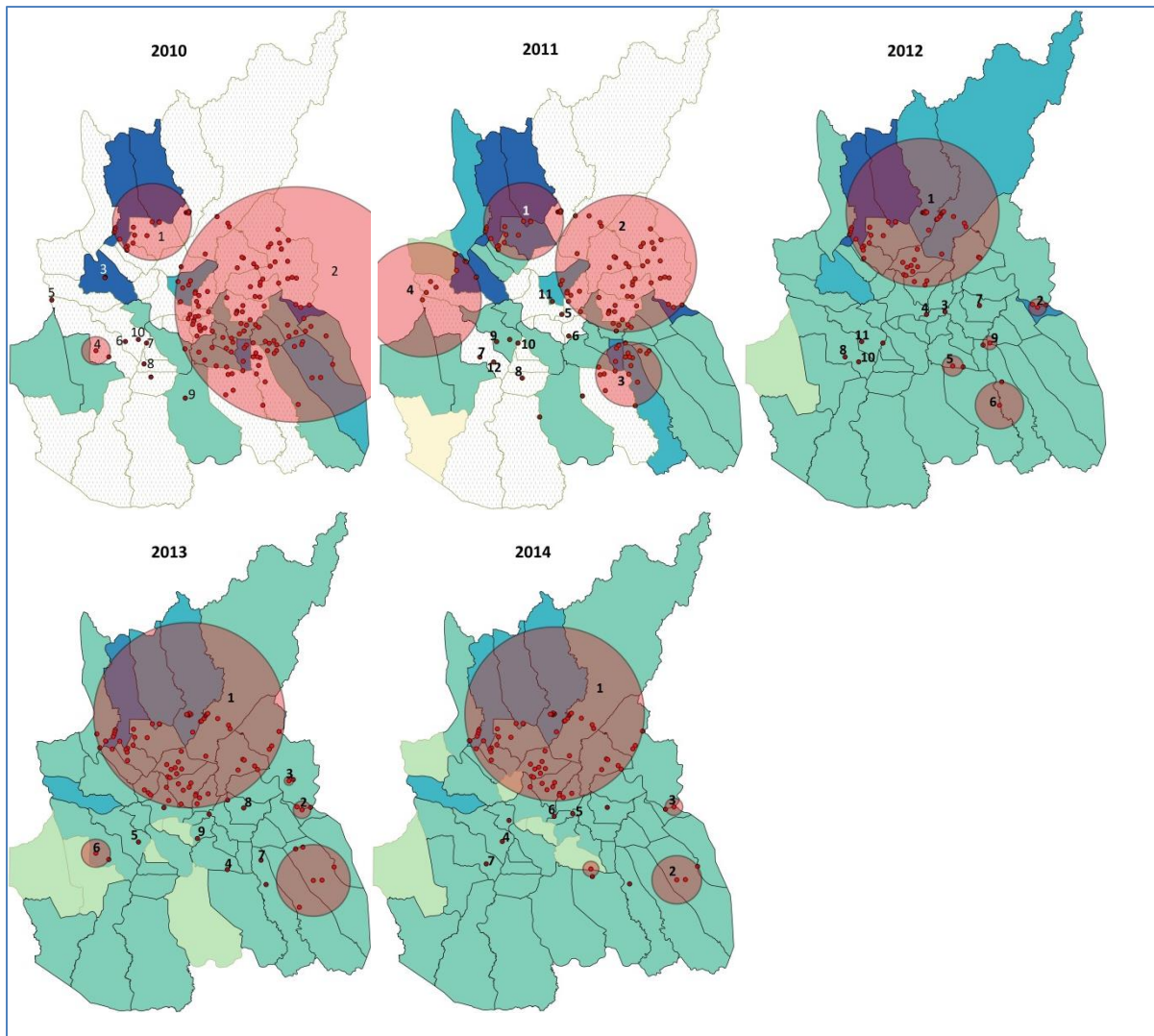

Spatial clusters of *Falciparum* malaria cases.

Supplement: Supplementary file 8 — Additional file 8. Spatial clusters of falciparum malaria cases. Clusters of villages with higher number of malaria cases detected from 2010 to 2014 in Ratanakiri Province. Each numbered circle or dot represents a significant cluster. The un-numbered circles or dots are not significant clusters. Incidence of falciparum malaria cases is given per commune. [file 12936_2017_1758_MOESM8_ESM.pdf]

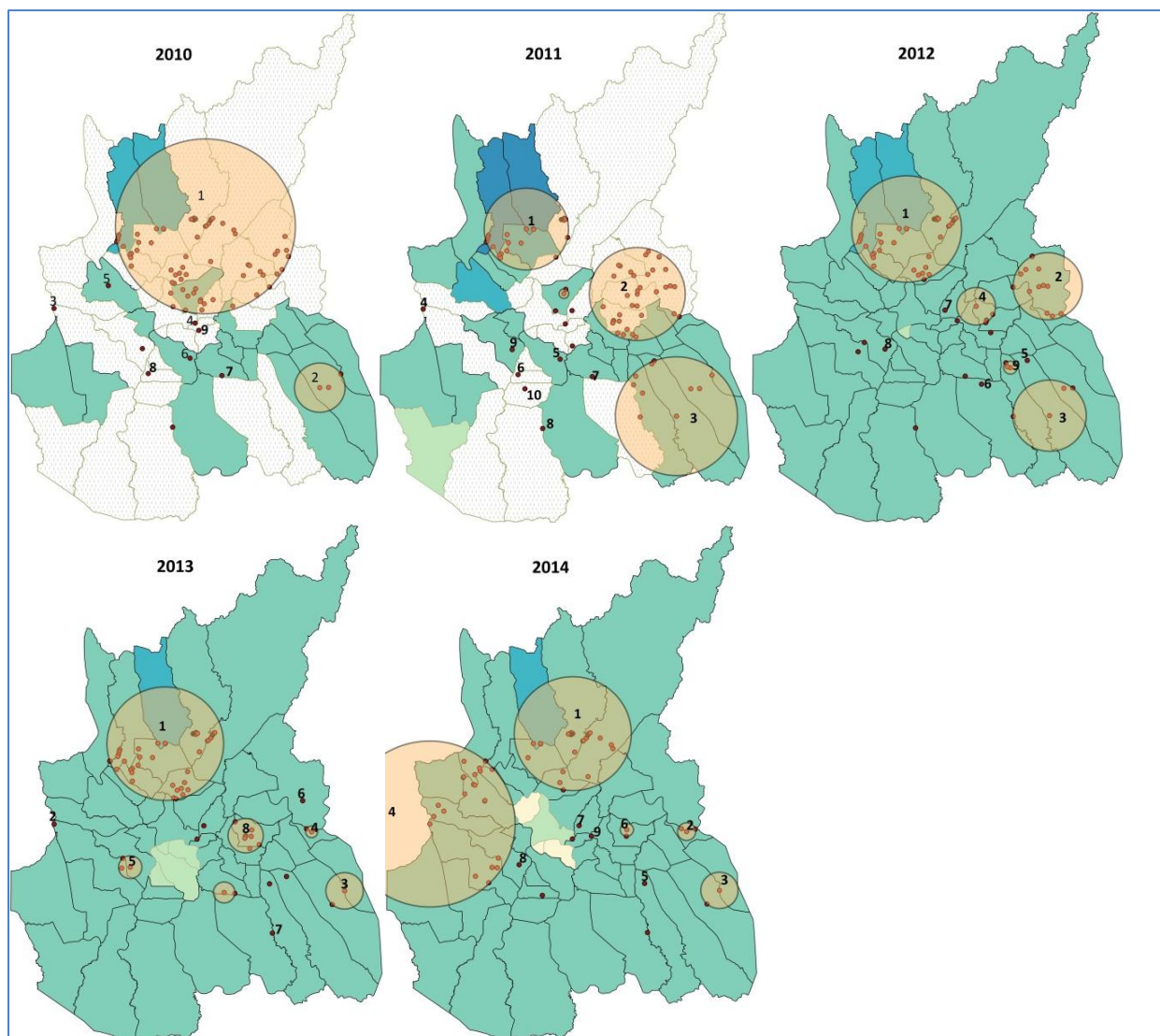

Spatial clusters of *Vivax* malaria cases.

Supplement: Supplementary file 10 — Additional file 10. Spatial clusters of vivax malaria cases. Clusters of villages with higher number of malaria cases detected from 2010 to 2014 in Ratanakiri Province. Each numbered circle or dot represents a significant cluster. The un-numbered circles or dots are not significant clusters. Incidence of vivax cases is given per commune. [file 12936_2017_1758_MOESM10_ESM.pdf]
