# Supplementary material for: Passive case detection of malaria in Ratanakiri Province (Cambodia) to detect villages at higher risk for malaria
Source: Malar J. 2017 Mar 6;16:104. doi: 10.1186/s12936-017-1758-3 (PMC5340042; doi:10.1186/s12936-017-1758-3)
Supplement: Supplementary file 4 — Additional file 4. Annual incidence rates per 1000 inhabitants for all reporting villages. The total number of reporting villages was 277 including main- and annex-villages, but not all of them reported every year. [file 12936_2017_1758_MOESM4_ESM.pdf]

Annual incidence rates per 1000 inhabitants for all reporting villages.

Year	No. villages reporting	Population	Incidence rate for all reporting villages	Median incidence rate (Q1 - Q3)
**All *Plasmodium* species**				
2010	174	109,734	73.76	70.66 (21.18 - 182.70)
2011	200	123,247	73.15	51.79 (13.80 - 165.90)
2012	263	165,537	39.94	31.37 (10.69 - 91.58)
2013	252	160,062	21.91	14.98 (5.89 - 45.61)
2014	226	148,710	25.25	16.94 (5.85 - 59.45)
P. falciparum				
2010	174	109,734	62.48	61.21 (11.85 - 154.90)
2011	200	123,247	55.05	41.84 (4.75 - 108.90)
2012	263	165,537	21.88	15.78 (5.15 - 37.86)
2013	252	160,062	11.14	5.81 (1.95 - 18.82)
2014	226	148,710	12.85	6.16 (1.59 – 21.56)
P. vivax				
2010	174	109,734	14.7	9.91 (2.00 – 29.63)
2011	200	123,247	19.79	10.56 (2.55 – 39.02)
2012	263	165,537	12.46	7.94 (2.15 – 23.88)
2013	252	160,062	7.39	4.63 (1.69 – 14.21)
2014	226	148,710	9.81	7.34 (2.71 – 18.19)
**Mixed infection**				
2010	174	109,734	1.72	0.00 (0.00 – 0.51)
2011	200	123,247	2.35	0.00 (0.00 – 2.67)
2012	263	165,537	4.68	1.30 (0.00 – 6.88)
2013	252	160,062	2.97	0.00 (0.00 – 3.81)
2014	226	148,710	4.15	0.71 (0.00 – 4.63)
