# Supplementary material for: Passive case detection of malaria in Ratanakiri Province (Cambodia) to detect villages at higher risk for malaria
Source: Malar J. 2017 Mar 6;16:104. doi: 10.1186/s12936-017-1758-3 (PMC5340042; doi:10.1186/s12936-017-1758-3)
Supplement: Supplementary file 6 — Additional file 6. Annual number of confirmed vivax malaria cases by village and vivax malaria incidence rate per 1000 inhabitants by commune. Although several villages did not report in 2010 and 2011 (37 and 28%, respectively), the number of confirmed malaria cases and incidence decreased steadily from 2012 to 2014. [file 12936_2017_1758_MOESM6_ESM.pdf]

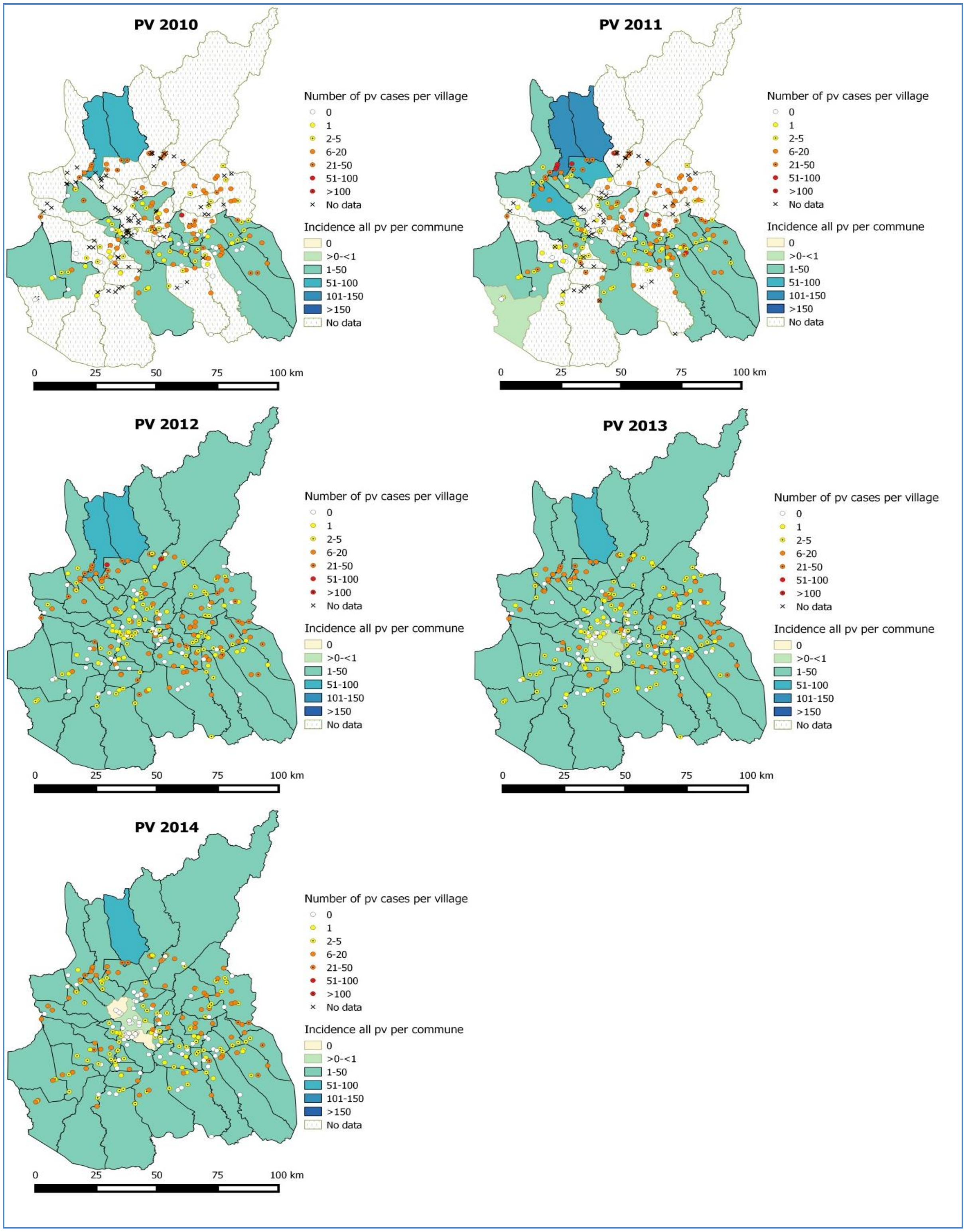


**Annual number of confirmed *Vivax* malaria cases by village and *Vivax* malaria incidence rate per 1000 inhabitants by commune.**
