# Supplementary material for: Passive case detection of malaria in Ratanakiri Province (Cambodia) to detect villages at higher risk for malaria
Source: Malar J. 2017 Mar 6;16:104. doi: 10.1186/s12936-017-1758-3 (PMC5340042; doi:10.1186/s12936-017-1758-3)
Supplement: Supplementary file 7 — Additional file 7. Spatial clusters of villages with significantly higher risk of all malaria cases (all Plasmodium species combined) from 2010 to 2014 in Ratanakiri Province. Only significant clusters are showed. RR: Relative risk. LLR: Log likelihood ratio. [file 12936_2017_1758_MOESM7_ESM.pdf]

Spatial clusters of villages with significant higher risk for all malaria cases (all *plasmodium* species combined) from 2010-2014 in Ratanakiri Province.

Year	Cluster	No. villages	Latitude	Longitude	Radius	Observed cases	Expected cases	RR	LLR	p-value
2010	1	21	14.046359	106.965239	13.01 km	1620	379	5.09	1218.22	<0.0001
2010	2	62	13.918318	107.275887	23.06 km	2402	1339	2.13	429.30	<0.0001
2010	3	7	13.489193	107.411705	17.76 km	387	94	4.26	259.11	<0.0001
2010	4	2	13.87759	106.82178	0.18 km	170	18	9.38	227.26	<0.0001
2010	5	2	13.606855	107.161185	3.43 km	183	27	7.03	198.27	<0.0001
2010	6	1	13.763939	107.081181	0 km	125	15	8.55	157.08	<0.0001
2010	7	4	13.661849	107.246883	4.16 km	234	73	3.26	112.84	<0.0001
2010	8	1	13.808123	106.658254	0 km	51	3	17.48	97.70	<0.0001
2010	9	1	13.658898	107.065608	0 km	133	30	4.55	97.11	<0.0001
2010	10	2	13.653405	106.793476	4.74 km	116	25	4.66	86.83	<0.0001
2010	11	1	13.676677	106.948091	0 km	76	18	4.36	53.13	<0.0001
2010	12	1	13.681226	106.883364	0 km	31	3	10.25	44.11	<0.0001
2010	13	1	13.61297	106.940432	0 km	62	17	3.75	36.30	<0.0001
2010	14	1	13.687627	106.923078	0 km	68	26	2.66	24.06	<0.0001
2010	15	1	13.508686	107.066478	0 km	45	22	2.04	9.12	0.0140
2010	16	1	13.751598	106.943513	0 km	40	20	2.04	8.13	0.0390
2011	1	25	14.046359	106.965239	13.01 km	2158	422	6.41	1974.11	<0.0001
2011	2	62	13.918318	107.275887	23.06 km	2697	1491	2.15	494.66	<0.0001
2011	3	20	13.582501	107.28396	10.85 km	921	477	2.04	174.07	<0.0001
2011	4	1	13.763939	107.081181	0 km	70	17	4.26	47.78	<0.0001
2011	5	2	13.698092	107.102015	0 km	91	29	3.11	41.28	<0.0001
2011	6	1	13.570719	106.960832	0 km	105	42	2.53	33.71	<0.0001
2011	7	1	13.681226	106.883364	0 km	22	3	6.52	22.60	<0.0001
2011	8	1	13.687627	106.923078	0 km	66	29	2.32	17.91	<0.0001
2011	9	1	13.802734	107.052193	0 km	51	20	2.57	16.87	<0.0001
2011	10	1	13.634391	106.832738	0 km	48	18	2.63	16.61	<0.0001
2011	11	1	13.452596	107.013707	0 km	82	43	1.93	14.28	0.0003
2011	12	1	13.61297	106.940432	0 km	2	19	2.38	12.60	0.0012
2011	13	1	13.733332	107.089609	0 km	51	24	2.12	11.29	0.0038
2011	14	1	13.676677	106.948091	0 km	42	20	2.15	9.69	0.0140
2012	1	49	14.045257	106.987059	19.15 km	2560	705	5.3	1775.32	<0.0001
2012	2	14	13.874754	107.406388	11.09 km	597	277	2.27	146.70	<0.0001
2012	3	1	13.772103	107.13765	0 km	53	8	6.76	55.97	<0.0001
2012	4	1	13.866858	107.082418	0 km	83	26	3.22	39.50	<0.0001
2012	5	5	13.489193	107.411705	13.33 km	125	55	2.29	32.88	<0.0001
2012	6	1	13.791049	107.242711	0 km	48	12	4.08	31.18	<0.0001
2012	7	2	13.606855	107.161185	3.43 km	64	22	2.97	27.06	<0.0001
2012	8	1	13.763939	107.081181	0 km	42	12	3.48	22.41	<0.0001
2012	9	1	13.707231	106.861432	0 km	61	26	2.36	17.17	<0.0001
2012	10	1	13.803495	107.101079	0 km	28	9	3.16	13.05	0.0005
2012	11	1	13.731991	107.379434	0 km	54	26	2.11	11.85	0.0015
2012	12	1	13.632246	107.295395	0 km	35	15	2.34	9.68	0.0110
2012	13	1	13.653769	107.345607	0 km	35	15	2.31	9.41	0.0120
2013	1	47	14.046359	106.965239	19.01 km	1519	376	6.36	1216.84	<0.0001
2013	2	3	13.785518	107.419929	2.90 km	108	30	3.71	61.77	<0.0001
2013	3	2	13.873367	107.380064	1.50 km	54	12	4.66	40.44	<0.0001
2013	4	1	13.60384	107.192836	0 km	31	5	6.39	31.25	<0.0001
2013	5	2	13.853864	107.076892	1.56 km	61	19	3.32	30.33	<0.0001
2013	6	7	13.67964	106.843052	6.10 km	141	72	2	26.66	<0.0001
2013	7	2	13.611548	107.517401	5.97 km	37	8	4.41	26.17	<0.0001
2013	8	1	13.687627	106.923078	0 km	36	11	3.26	17.51	<0.0001
2013	9	1	13.791049	107.242711	0 km	25	6	4.01	15.90	<0.0001
2013	10	1	13.632246	107.295395	0 km	28	8	3.54	15.24	0.0001
2014	1	41	14.046359	106.965239	18.13 km	1123	339	4.3	658.33	<0.0001
2014	2	7	13.681226	106.883364	4.36 km	315	93	3.62	170.14	<0.0001
2014	3	3	13.57074	107.454313	8.19 km	120	19	6.34	119.13	<0.0001
2014	4	3	13.785518	107.419929	2.90 km	135	32	4.35	93.10	<0.0001
2014	5	1	13.772103	107.13765	0 km	31	4	6.96	33.52	<0.0001
2014	6	2	13.74949	106.954309	1.19 km	54	15	3.75	31.57	<0.0001
2014	7	1	13.632246	107.295395	0 km	36	9	4.26	24.50	<0.0001
2014	8	1	13.866858	107.082418	0 km	48	15	3.27	23.45	<0.0001
2014	9	1	13.791049	107.242711	0 km	26	7	3.89	15.97	0.0001
2014	10	1	13.803495	107.101079	0 km	21	5	4.18	14.02	0.0003
2014	11	1	13.763939	107.081181	0 km	22	7	3.21	10.49	0.0140
2014	12	1	13.789543	107.307792	0 km	29	11	2.55	9.47	0.0210
