# Supplementary material for: Passive case detection of malaria in Ratanakiri Province (Cambodia) to detect villages at higher risk for malaria
Source: Malar J. 2017 Mar 6;16:104. doi: 10.1186/s12936-017-1758-3 (PMC5340042; doi:10.1186/s12936-017-1758-3)
Supplement: Supplementary file 9 — Additional file 9. Spatial clusters of villages with significantly higher risk of falciparum malaria cases from 2010 to 2014 in Ratanakiri Province. Only significant clusters are showed. RR: Relative risk. LLR: Log likelihood ratio. [file 12936_2017_1758_MOESM9_ESM.pdf]

Spatial clusters of villages with significant higher risk for *Falciparum* malaria cases from 2010-2014 in Ratanakiri Province.

Year	Cluster	No. villages	Latitude	Longitude	Radius	Observed cases	Expected cases	RR	LLR	p-value
2010	1	21	14.046359	106.965239	13.01 km	1302	300	5.19	995.96	<0.0001
2010	2	131	13.794314	107.404628	39.92 km	3637	2580	1.95	353.24	<0.0001
2010	3	2	13.87759	106.82178	0.18 km	143	15	9.98	198.98	<0.0001
2010	4	2	13.653405	106.793476	4.74 km	111	20	5.64	100.09	<0.0001
2010	5	1	13.808123	106.658254	0 km	30	2	12.96	49.12	<0.0001
2010	6	1	13.681226	106.883364	0 km	29	2	12.11	45.67	<0.0001
2010	7	1	13.676677	106.948091	0 km	62	14	4.49	44.77	<0.0001
2010	8	1	13.61297	106.940432	0 km	49	13	3.74	28.62	<0.0001
2010	9	1	13.508686	107.066478	0 km	44	18	2.52	14.11	0.0002
2010	10	1	13.687627	106.923078	0 km	44	20	2.17	10.36	0.0054
2011	1	21	14.046359	106.965239	13.01 km	1570	301	6.58	1465.65	<0.0001
2011	2	62	13.918318	107.275887	23.06 km	2005	1064	2.29	417.68	<0.0001
2011	3	20	13.582501	107.28396	10.85 km	705	340	2.21	160.33	<0.0001
2011	4	11	13.808123	106.658254	19.26 km	315	162	2	58.52	<0.0001
2011	5	1	13.763939	107.081181	0 km	59	12	5.05	48.02	<0.0001
2011	6	2	13.698092	107.102015	0 km	75	21	3.6	41.64	<0.0001
2011	7	1	13.634391	106.832738	0 km	48	13	3.69	27.63	<0.0001
2011	8	1	13.570719	106.960832	0 km	78	30	2.64	26.99	<0.0001
2011	9	1	13.681226	106.883364	0 km	19	2	7.9	22.65	<0.0001
2011	10	1	13.676677	106.948091	0 km	38	14	2.73	14.08	0.0002
2011	11	1	13.802734	107.052193	0 km	37	14	2.61	12.63	0.0009
2011	12	1	13.619313	106.874335	0 km	21	7	2.88	8.48	0.0390
2012	1	53	14.074678	107.069142	25.14 km	1470	421	5.13	978.52	<0.0001
2012	2	3	13.785518	107.419929	2.90 km	155	32	5.09	125.58	<0.0001
2012	3	1	13.772103	107.13765	0 km	45	4	10.28	64.00	<0.0001
2012	4	1	13.763939	107.081181	0 km	42	7	6.24	41.48	<0.0001
2012	5	2	13.606855	107.161185	3.43 km	51	12	4.24	34.45	<0.0001
2012	6	2	13.486845	107.303896	7.97 km	40	11	3.61	22.30	<0.0001
2012	7	1	13.791049	107.242711	0 km	28	7	4.25	19.03	<0.0001
2012	8	1	13.634391	106.832738	0 km	26	8	3.47	13.79	0.0003
2012	9	2	13.677142	107.274104	2.32 km	43	19	2.24	10.78	0.0041
2012	10	1	13.619313	106.874335	0 km	16	4	3.8	9.57	0.0160
2012	11	1	13.681226	106.883364	0 km	9	1	6.48	9.21	0.0200
2013	1	80	14.072846	107.077263	31.30 km	1008	342	5.36	602.51	<0.0001
2013	2	3	13.785518	107.419929	2.90 km	73	15	4.89	56.79	<0.0001
2013	3	2	13.873367	107.380064	1.50 km	41	6	6.88	43.69	<0.0001
2013	4	1	13.60384	107.192836	0 km	22	3	8.78	28.19	<0.0001
2013	5	1	13.687627	106.923078	0 km	28	6	4.92	22.18	<0.0001
2013	6	2	13.653405	106.793476	4.74 km	26	6	4.65	19.43	<0.0001
2013	7	1	13.632246	107.295395	0 km	21	4	5.14	17.39	<0.0001
2013	8	1	13.791049	107.242711	0 km	15	3	4.65	11.23	0.0030
2013	9	2	13.698092	107.102015	0 km	19	6	3.22	9.08	0.0230
2014	1	71	14.075614	107.082241	29.43 km	894	293	5.07	535.37	<0.0001
2014	2	3	13.57074	107.454313	8.19 km	73	9	8.11	87.67	<0.0001
2014	3	2	13.792797	107.445777	2.90 km	54	10	5.62	48.25	<0.0001
2014	4	1	13.687627	106.923078	0 km	36	6	6.42	36.28	<0.0001
2014	5	1	13.772103	107.13765	0 km	22	2	10.35	31.43	<0.0001
2014	6	1	13.763939	107.081181	0 km	14	3	4.27	9.57	0.0100
2014	7	1	13.619313	106.874335	0 km	11	2	5.4	9.56	0.0110
