# Supplementary material for: Passive case detection of malaria in Ratanakiri Province (Cambodia) to detect villages at higher risk for malaria
Source: Malar J. 2017 Mar 6;16:104. doi: 10.1186/s12936-017-1758-3 (PMC5340042; doi:10.1186/s12936-017-1758-3)
Supplement: Supplementary file 11 — Additional file 11. Spatial clusters of villages with significantly higher risk of vivax malaria cases from 201 to 2014 in Ratanakiri Province. Only significant clusters are showed. RR: Relative risk. LLR: Log likelihood ratio. [file 12936_2017_1758_MOESM11_ESM.pdf]

Spatial clusters of villages with significant higher risk for *Vivax* malaria cases from 2010-2014 in Ratanakiri Province.

Year	Cluster	No. villages	Latitude	Longitude	Radius	Observed cases	Expected cases	RR	LLR	p-value
2010	1	81	14.053707	107.11178	29.06 km	705	285	3.77	301.22	<0.0001
2010	2	3	13.57074	107.454313	8.19 km	77	8	10.33	108.60	<0.0001
2010	3	1	13.808123	106.658254	0 km	21	1	38.94	56.30	<0.0001
2010	4	1	13.763939	107.081181	0 km	34	3	12.57	54.44	<0.0001
2010	5	1	13.876213	106.82098	0 km	26	2	12.8	42.12	<0.0001
2010	6	1	13.658898	107.065608	0 km	37	6	6.85	39.28	<0.0001
2010	7	1	13.606855	107.161185	0 km	20	3	7.11	21.94	<0.0001
2010	8	1	13.61297	106.940432	0 km	13	3	4.22	8.76	0.0200
2010	9	1	13.742276	107.091971	0 km	8	1	6.71	8.41	0.0270
2011	1	25	14.046359	106.965239	13.51 km	606	131	5.89	507.52	<0.0001
2011	2	40	13.85282	107.295247	15.39 km	532	259	2.37	129.22	<0.0001
2011	3	10	13.489193	107.411705	19.63 km	172	53	3.42	86.47	<0.0001
2011	4	1	13.808123	106.658254	0 km	24	1	28.93	57.47	<0.0001
2011	5	1	13.658898	107.065608	0 km	32	8	3.83	19.18	<0.0001
2011	6	1	13.61297	106.940432	0 km	22	5	4.66	16.53	<0.0001
2011	7	1	13.606855	107.161185	0 km	21	4	4.85	16.42	<0.0001
2011	8	1	13.452596	107.013707	0 km	34	11	3.14	15.59	0.0001
2011	9	1	13.687627	106.923078	0 km	22	7	3.02	9.57	0.0110
2011	10	1	13.570719	106.960832	0 km	27	11	2.54	8.72	0.0320
2012	1	40	14.045257	106.987059	17.60 km	694	176	5.39	510.99	<0.0001
2012	2	15	13.874754	107.406388	11.10 km	212	91	2.47	61.70	<0.0001
2012	3	4	13.489193	107.411705	11.79 km	55	13	4.3	37.63	0.0000
2012	4	5	13.815022	107.193675	6.21 km	63	22	2.89	25.22	0.0000
2012	5	1	13.653769	107.345607	0 km	26	5	5.41	22.59	0.0000
2012	6	1	13.583191	107.209723	0 km	18	4	4.11	11.78	0.0016
2012	7	1	13.803495	107.101079	0 km	13	3	4.6	9.65	0.012
2012	8	1	13.687627	106.923078	0 km	20	7	3.01	8.63	0.024
2012	9	3	13.632246	107.295395	2.15 km	27	11	2.51	8.57	0.027
2013	1	47	14.045257	106.987059	18.58 km	473	124	5.62	347.31	<0.0001
2013	2	1	13.808123	106.658254	0 km	20	0	46.53	57.07	<0.0001
2013	3	2	13.611548	107.517401	5.97 km	31	3	10.92	45.61	<0.0001
2013	4	2	13.785518	107.419929	1.92 km	24	5	4.45	17.08	<0.0001
2013	5	3	13.681226	106.883364	3.74 km	36	14	2.65	12.42	0.0009
2013	6	1	13.877276	107.393375	0 km	11	2	7.18	12.18	0.0011
2013	7	1	13.486845	107.303896	0 km	8	1	7.8	9.44	0.0120
2013	8	8	13.774297	107.224708	5.63 km	45	22	2.07	9.21	0.0160
2014	1	27	14.075614	107.082241	18.72 km	220	64	3.87	124.13	<0.0001
2014	2	3	13.785518	107.419929	2.90 km	68	12	6.08	64.69	<0.0001
2014	3	2	13.611548	107.517401	5.97 km	40	3	12.4	63.42	<0.0001
2014	4	24	13.808123	106.658254	27.30 km	216	101	2.36	54.77	<0.0001
2014	5	1	13.632246	107.295395	0 km	24	3	7.82	28.26	<0.0001
2014	6	2	13.791049	107.242711	2.11 km	29	7	4.26	19.67	<0.0001
2014	7	1	13.803495	107.101079	0 km	13	2	7.1	14.26	0.0001
2014	8	1	13.687627	106.923078	0 km	16	4	3.71	9.23	0.0170
2014	9	1	13.772103	107.13765	0 km	9	2	5.52	7.98	0.0460
